# Celastrol restricts experimental periodontitis related alveolar bone loss by suppressing inflammatory cytokine response

**DOI:** 10.37796/2211-8039.1421

**Published:** 2023-12-01

**Authors:** Ahmet Altın, Meltem Zihni Korkmaz, Mehtap Atak, Tolga Mercantepe, Hülya Kılıç Yılmaz

**Affiliations:** a Istanbul Kent University, Faculty of Dentistry, Department of Periodontology, İstanbul, Turkey; b Recep Tayyip Erdogan University, Faculty of Dentistry, Department of Periodontology, Rize, Turkey; c Recep Tayyip Erdogan University, Faculty of Medicine, Department of Biochemistry, Rize, Turkey; d Recep Tayyip Erdogan University, Faculty of Medicine, Department of Histology and Embryology, Rize, Turkey

**Keywords:** Periodontitis, Alveolar bone loss, Celastrol, Tripterygium, Terpene, Anti-inflammatory agents, Tumor necrosis factor-alpha, Interleukin-1beta

## Abstract

**Introduction:**

Periodontitis is a common chronic inflammatory disease characterized by the destruction of the supporting structures of the teeth. The host defense mechanisms are responsible for inflamatuar and destructive reactions in periodontitis. Celastrol is one of the most promising components of the plant in Eastern and Southern China that has a long history of use in traditional medicine for the treatment of inflammatory conditions.

**Aim:**

The aim of this animal study was to inspect the preventive or restrictive effects of celastrol on periodontitis-related inflammatory host response and alveolar bone loss.

**Methods:**

24 male Sprague Dawley rats were randomly assigned into 3 groups: control, experimental periodontitis (Ep), and experimental periodontitis-celastrol (Ep-Cel). Periodontitis was induced by placing ligatures sub-paramarginally around the mandibular first molars of the rats in the Ep and Ep-Cel groups and maintaining the ligatures for 15 days. For 14 days following the ligature placement, celastrol administration (1 mg/kg BW day) for the Ep-Cel group and vehicle injection for the control and Ep groups was carried out. At the end of the experiment, mandibula and gingiva samples were obtained after the euthanasia. Alveolar bone loss was measured on serial histological slices; Tumor Necrosis Factor-α and Interleukin-1β levels were measured on gingiva samples by ELISA.

**Results:**

Systemic celastrol administration significantly restricted the alveolar bone loss that was higher in rats with periodontitis. (p < 0.05) Tumor Necrosis Factor-α and Interleukin-1β levels that were high in the gingiva of the rats with periodontitis were found significantly lower in rats administered celastrol. (p < 0.05)

**Conclusion:**

Celastrol restricted periodontitis-related alveolar bone loss by suppressing the inflammatory response.

## 1. Introduction

Periodontitis, a globally prevalent chronic inflammatory disease characterized by the destruction of the supporting structures of the teeth, has negative effects on the quality of life with tooth loss, impaired function, poor aesthetics, and worsened systemic health status [1,2]. Although dental plaque has long been regarded as the principal etiologic factor for periodontitis, current literature suggests that the destructive clinical condition is the result of the host defense mechanism in response to periodontopathogens [3,4]. The knowledge of this key role of the host defense in periodontal tissue destruction has encouraged the search for adjuncts to the conventional treatment of periodontitis which covers the professional elimination of plaque, calculus, and plaque retentive factors and motivation of the patient for better oral hygiene management. Non Steroidal Antiinflammatory Drugs (NSAIDs), bisphosphonates, probiotics, and other several agents have been researched in the context of limiting the destructive aspects of the host response while promoting the constructive and regulatory aspects. Currently, sub-antimicrobial dose doxycycline is the only systemically administered host modulator agent approved by the U.S. Food and Drug Administration (FDA) specifically for the treatment of chronic periodontitis, therefore further research for new agents is required [5–7].

Celastrol, also called tripterine is one of the most promising components of the plant.

*Tripterygium wilfordii* Hook. f. (Celastraceae), a perennial vine native to Eastern and Southern China. In these regions, the extracts of this plant have a long history of use in traditional medicine for the treatment of swelling, fever, sores, joint pain, and other inflammatory conditions [8]. Celastrol shows its pharmacological activity via forming covalent protein adducts by reacting with thiol groups of cysteine residues of regulatory proteins. The main mechanism behind the antiinflammatory activity of celastrol is through inhibition of inhibitor of kappa B kinase (IKK)-nuclear factor kappa B (NF-κB) signaling. Celastrol suppresses NF-kB activation by inhibiting IKK activity, therefore, inhibits the expression of various pro-inflammatory mediators [9–11]. Luk et al. [12] demonstrated that *Tripterygium wilfordii* extracts modulate the inflammatory response by suppressing lipopolysaccharide (LPS) induced TNF-α (tumor necrosis factor-α) gene expression on cell culture. Gao et al. [13] found that celastrol administration reduces the serum levels of TNF-α, Interleukin-1β (IL-1β), Interleukin 6 (IL-6), and Interferon-gamma (IFN-γ) that were increased in a collagen-induced arthritis model. In China, a patent drug prepared from Tripterygium Wilfordii extract called tripterygium glycoside tablet is used to treat autoimmune diseases, such as rheumatoid arthritis and nephrotic syndrome [14]. To the extent of our knowledge, this study is the first to evaluate the effects of celastrol on periodontal destruction.

In light of this information, the present preliminary study was designed with the hypothesis that systemic celastrol administration may be a helpful adjunctive therapeutic agent in the prevention or restriction of periodontal tissue destruction. Furthermore, it is to be hoped that this study will play a pivotal role for further studies in research for cost-effective, natural preventive modalities in the management of destructive forms of periodontal disease.

## 2. Materials and methods

### 2.1. Ethical approval, animal housing, and study groups

The experimental protocol of this study was approved by the Local Ethical Committee for Animal Experiments of Recep Tayyip Erdogan University (2019/19). Experimental procedures were carried out at Recep Tayyip Erdogan University Experimental Animals.

Application and Research Center with human care as specified in the “Guide for the Care and Use of Laboratory Animals” prepared by the National Academy of Sciences and published by the National Institutes of Health. 24 male Spraque dawley rats (age: 3–4 months, weight range 320 ± 30 g) were obtained from the same facility, sheltered under standard laboratory conditions (light period: 6:00 a.m. – 7:00 p.m.; temperature: 22 ± 2 °C; and relative humidity: 58%) and fed with standard rat chow and water during the study. The general health status of the animals was observed by a veterinarian of the facility throughout the experiment. The rats were randomly assigned into 3 groups: control, experimental periodontitis (Ep), and experimental periodontitis-celastrol (Ep-Cel).

### 2.2. Induction of periodontitis

After 7 days of adaptation period, the rats in the Ep and Ep-Cel groups were anesthetized by intraperitoneal (IP) injections of xylazine hydrochloride^1^ (10 mg/BW) and ketamine hydrochloride^2^ (40 mg/BW) Periodontitis was induced by placing 4.0 silk sutures sub-para-marginally around the necks of right and left mandibular first molars and maintaining the sutures in place until the day of sampling. The silk suture with its braided, multifilament, and non-absorbable properties causes inflammatory changes by its presence and plaque accumulation and results in periodontitis [15–17].

### 2.3. Celastrol administration

On the next day following the periodontitis induction, the celastrol administration was began. Celastrol in crystalline solid form^3^ was obtained. Daily total doses were calculated to match 1 (mg/kg BW/day). The total dosage for each day was taken separately into eppendorf tubes and stored at −20 °C. On the morning of each administration day, the daily dosage was taken from the freezer. The stock solution was prepared by adding dimethyl sulfoxide^4^ (DMSO) to celastrol in the eppendorf tube. Then, the stock solution was diluted with phosphate-buffered saline (PBS) (pH:7.2) to prepare the final injectable solution. The prepared celastrol solution was administered to the rats in the Ep-Cel group intraperitoneally (IP) at 8:00–8:30 am. in the morning for 14 days. The rats in the Ep and control groups were injected with only the vehicle (DMSO + PBS).

### 2.4. Tissue sampling

On the next day after the 14th administration, all rats were anesthetized with the same medications detailed in the induction of periodontitis. The rats were then euthanized with a lethal injection of pentobarbital (150 mg/kg). After confirmation of humane death, mandibular samples were dissected. The right mandibular samples were transferred into 10% neutral formaldehyde solution and spared for morphometric analysis. 2–3 mm. wide gingiva samples were carefully excised from around the cervix of first molars of left mandibular samples, taken into separate eppendorf tubes, and forwarded to biochemical analysis.

### 2.5. Determination of alveolar bone levels

**Fig. 1:**
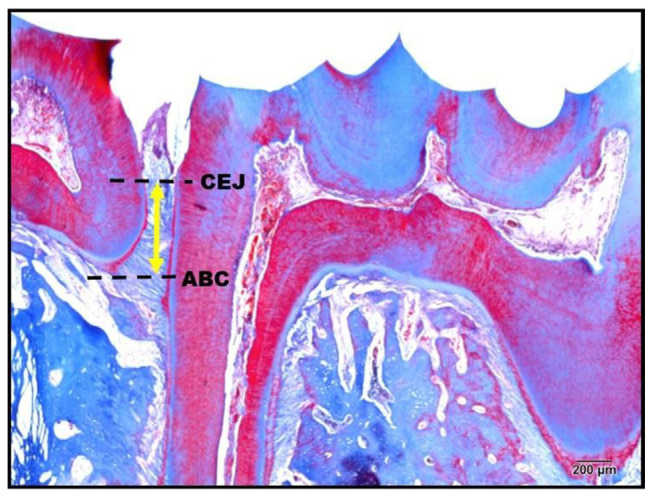
Histomorphometric measurement of the distance between cemento-enamel junction (**CEJ**) and alveolar bone crest (**ABC**).

Bone losses on the distal sides of the right mandibular first molar teeth were measured histomorphometrically. The samples were fixed in 10% neutral buffered formaldehyde for 48 h and then decalcified with 5% nitric acid solution at room temperature for 7 days. The nitric acid solution was changed every 24 h and on the last days, decalcification was tested with a needle. The decalcified mandibular samples were then neutralized with sodium bicarbonate for 1 h and after dehydration, embedded in paraffin, and serially sectioned on the mesiodistal plane in 5 μm-thickness using a microtome^5^. Then, the slices were stained with Masson trichrome.^6^ Randomly chosen ten slices per rat were used for the measurement of the distance between the cementoenamel junction (CEJ) and the alveolar bone crest (ABC) with a trinocular light microscope^7^ attached to a digital camera^8^ and an analyzing software^9^ (Fig. 1) The results were expressed as mean ± standard deviation (SD) (μm).

### 2.6. Determination of gingival TNF-α and IL-1β levels

Rat-specific ELISA kits were used to determine levels of TNF-α^10^ and IL-1 β^11^ in line with the manufacturer’s protocols. The results were expressed as mean ± Standard Deviation (SD) (pg/mg prt) of the concentration of each factor.

### 2.7. Statistical analysis

All data were calculated as mean and SD using a statistical software^12^. The normal distribution was confirmed by Shapiro–Wilk and Kolmogorov–Smirnov tests. The data were analyzed with one-way analysis of variance (ANOVA) and Tukey’s honestly significant difference tests. p < 0.05 at a confidence interval of 95% was regarded as statistically significant.

## 3. Results

### 3.1. Morphometric results

**Fig. 2:**
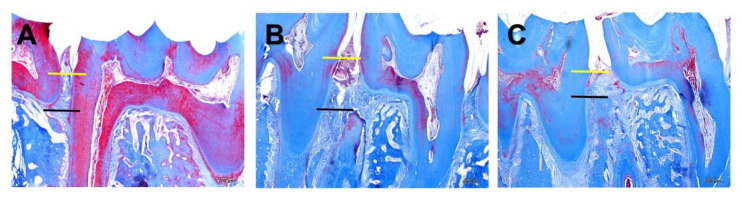
Histological illustrations of mesiodistal histological slices of mandibular first molar region. **A)** Control group section **B)** Ep group section C) Ep-Cel group section Yellow line: level of cemento-enamel junction; Black line: level of alveolar bone crest.

Table 1 and Fig. 2 show the comparison of morphometric measurements between the groups.

CEJ-ABC distance measurements of the Ep group were significantly higher, compared with the control group. (p < 0.05) The same measurements of the Ep-Cel group were significantly lower, compared with the Ep group. (p < 0.05).

### 3.2. Biochemical results

Table 2 shows the comparison of gingival levels of TNF-α and IL-1β between the groups.

Gingival TNF-α level was significantly higher in the Ep group, compared with the control group. (p < 0.05) It was significantly lower in the Ep-Cel group, compared with the Ep group (p < 0.05) while being higher than the control group.

Gingival IL-1β level was significantly higher in the Ep group, compared with the control group. (p < 0.05) It was significantly lower in the Ep-Cel group, compared with the Ep group (p < 0.05) while being higher than the control group.

## 4. Discussion

The present study shows that systemic celastrol administration suppresses experimental periodontitis-related gingival inflammation and alveolar bone loss. Periodontitis is an inflammatory destructive disease of the tooth-supporting tissues. Although dental plaque is the primary etiological factor, the host response to microbial attack accounts for the majority of periodontal tissue destruction [1,3,18]. Scaling and root planing by mechanical means is considered the standard approach for the treatment of periodontal diseases, primarily in order to remove the etiological factor. However, given the complex nature of the disease, the diversity of risk factors, and the fact that the non-specific plaque hypothesis is no longer supported today, new approaches are required to support conventional methods. In this context, there is promise in host modulation therapies by which the host response is regulated, constructive and protective aspects are supported whereas destructive aspects are suppressed [5,6].

Rodent models have been important contributors to the understanding of molecular mechanisms in the pathogenesis of inflammatory diseases [19]. Since the present study is the first to evaluate the potential effects of celastrol on periodontal destruction, comparative testing of the hypothesis was imperative to be carried on an experimental model. Creating and increasing areas of plaque retention by applying ligatures around the molars of rats to induce periodontitis is a successful method that has been tried and tested in previous studies [15,16]. Kuhr et al. [17] concluded that the periodontitis model induced by ligature shows its pronounced effects by an acute course in a limited time and suggested that this model is best suitable for experiments that last 15 days or less. Since the main scope of the present study was the evaluation of the preventive effects of celastrol on periodontal inflammation and tissue destruction, 14 days of ligature application was preferred and celastrol administration was began on the first day following the suturing.

Celastrol is a bioactive molecule with anti-inflammatory, immunosuppressive, antioxidant, and anticancer effects via interaction with various regulatory proteins in the body. In this context, the effect of celastrol as a supportive agent for the treatment of inflammatory diseases and conditions, especially rheumatoid arthritis, has been investigated and promising results have been reached [10,11]. In an adjuvant arthritis model, Li et al. [20] showed anti-arthritic effect of celastrol by limiting edema and bone destruction. Gao et al. [13] found that celastrol reduced inflammatory and oxidative stress and improved edema and arthritis scores in rats with collagen-induced arthritis. In a double-blind placebo-controlled study, Tao et al. [21] showed that Trypterygium wilfordii extract is successful in the treatment of rheumatoid arthritis according to the criteria of the American Society of Rheumatology and is well tolerated by patients in therapeutic doses. Due to similarities in pathogenetic mechanisms with chronic inflammatory and autoimmune diseases and conditions such as Crohn’s disease, rheumatoid arthritis, ankylosing spondylitis, psoriasis, and ulcerative colitis, biological anti-cytokine treatments for these conditions were also seen as promising for periodontal treatment [6,22]. In both rheumatoid arthritis and periodontitis, there is a pronounced irregularity in inflammatory cytokine pathways, and immunogenetic risk factors concerning IL-1, TNF, and Prostaglandin E2 expression are common, therefore therapeutic approaches targeting pathogenetic mechanisms of chronic inflammatory diseases like rheumatoid arthritis are valuable for periodontal research [6,23].

Unfortunately, there is no current agreement regarding the dose and duration of the Celastrol application. In studies accessed by our team [24–26], the daily dose for rats was dominantly 1 mg/kg BW, and the period of administration varied between 10 and 28 days. In an experimental arthritis study with 1 mg/kg BW celastrol administration for 28 days, Gao et al. [13] found that edema was significantly suppressed from day 14. Since the present study is the first celastrol administration in the field of periodontology, considering the main aim of evaluating the protective and supportive effects and in light of the aforementioned literature review, 1 mg/kg IP administration for the first 14 days of ligature placement with the maximum acute inflammatory and destructive activity was preferred.

Alveolar bone destruction is the most characteristic aspect of inflammatory periodontal disease [27]. In the present study, alveolar bone destruction related to experimental periodontitis was observed in rats with only ligatures. Though being present in the Ep-Cel group, periodontitis-related alveolar bone destruction was found to be statistically significantly lower compared with the Ep group. This result, which is also supported by the biochemical results, suggests that systemic anti-inflammatory application may restrict periodontitis-related alveolar bone destruction and that celastrol may be a potential adjunctive therapeutic for preventing or mitigating the effects of destructive periodontal disease.

The rationale for evaluating levels of TNF-α and IL-1β in this preliminary study was that these frequently studied cytokines play key roles in inflammatory periodontal destruction, and inhibition of these cytokines ameliorates the disease activity [3,28,29]. In several animal and cell culture studies [13,30–32] celastrol and other *Tripterygium wilfordii* extracts were shown to restrict pathological TNF-α and IL-1β expression. Luk et al. [12] found that Tripterygium Wilfordii extract limits the LPS induced increase in TNF-α and IL1β levels. In the present study, it was found that there was an increase in TNF-α and IL-1β levels in rat gingiva after periodontitis induction and systemic celastrol application suppressed this increase.

This biochemical finding is consistent with the current literature and helpful in confirming the anti-inflammatory role of celastrol in preventing periodontitis-related alveolar bone destruction. The inflammatory cytokines IL-1 and TNF-α and induction of osteoclastic differentiation play a major role in the progression of periodontal bone destruction [33]. The NF-κB, as a transcription factor, is important in the production of various inflammatory mediators including but not limited to IL-1 and TNF-α. Furthermore, receptor activator of NF-κB ligand (RANKL)-induced NF-κB activity in osteoclast precursors plays a role in osteoclastic differentiation [34,35]. Inhibition of the NF-κB pathway by celastrol [9,36] and othermolecules [35] has been shown to ameliorate inflammatory bone diseases. In this context, the inhibitory effect of celastrol on the NF-κB pathway may have been a factor behind the biochemical and morphometric results of the present study.

This study has some limitations. It may be misleading to directly associate the acute periodontal tissue destruction observed in experimental periodontitis with human periodontitis. Moreover, the findings we have obtained only on male rats may not fully reflect the entire population. Although TNF-α and IL-1β are key cytokines of the inflammatory host response, additional parameters could not be investigated due to the limited amount of gingival tissue samples. The absence of a consensus on the dosage and method of celastrol administration in the treatment of both periodontitis and other diseases necessitates further comprehensive studies on this topic. In order to understand the effects of celastrol on periodontal tissues, more comprehensive experimental studies, cell culture studies, and microbiology-based studies are required. In addition, studies with different celastrol dosages will provide clearer data to evaluate both the limiting effect on the induction of periodontitis and the therapeutic effect after periodontitis induction.

## 5. Conclusion

It can be concluded that systemic celastrol administration restricts periodontal inflammation and related alveolar bone loss. Based on this research, further studies about the mechanism and therapeutic properties of celastrol might contribute to its clinical application in the future.

## Figures and Tables

**Table 1:** Comparison of the alveolar bone levels between the groups.

	Control (n:8) mean ± SD	Ep (n:8) mean ± SD	Ep-Cel (n:8) mean ± SD
**CEJ-ABC (μm)**	1025.50 ± 100.64^a^	1449.98 ± 346.29^b^	1060.26 ± 381.38^c^

Different superscript letters indicate a significant difference between the groups (p < 0.05); SD: standard deviation.

**Table 2:** Comparison of the gingival Levels of TNF-α and IL-1β.

	Control (n:8) mean ± SD	Ep (n:8) mean ± SD	Ep-Cel (n:8) mean ± SD
**TNF-α (pg/mg prt)**	137 ± 12^a^	280 ± 42 ^b^	238 ± 30 ^c^
**IL-1β (pg/mg prt)**	75 ± 29^a^	124 ± 41^b^	80 ± 6^a^

Different superscript letters indicate a significant difference between the groups (p < 0.05); SD: standard deviation.

## Abbreviations

IKK: Inhibitor of kappa B kinase
NF-κB: Nuclear factor kappa B
TNF-α: Tumor necrosis factor-α
IL-1β: Interleukin-1β
